# Supplementary material for: The cost of sleeping sickness vector control in Yasa Bonga, a health district in the Democratic Republic of the Congo
Source: PLoS Negl Trop Dis. 2024 Nov 21;18(11):e0011959. doi: 10.1371/journal.pntd.0011959 (PMC11666030; doi:10.1371/journal.pntd.0011959)
Supplement: S1 Annex — I. Illustrations of vector control, II. Calendar of vector control activities between 2015 and 2017, III. Details Financial costs, IV. Details Economic costs. (DOCX) [file pntd.0011959.s001.docx]

S1 Annex

Illustrations of vector control in the DRC

Fig A. Installation of a Tiny Target on a riverbank ((c) Inaki Tirados, LSTM)


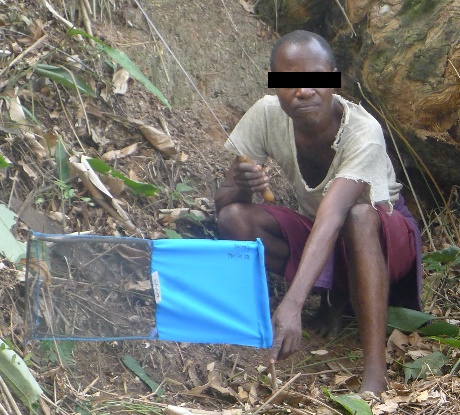


Fig B. Deployment Tiny Targets by canoe ((c) Inaki Tirados, LSTM)


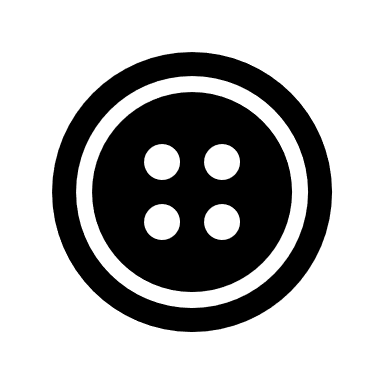

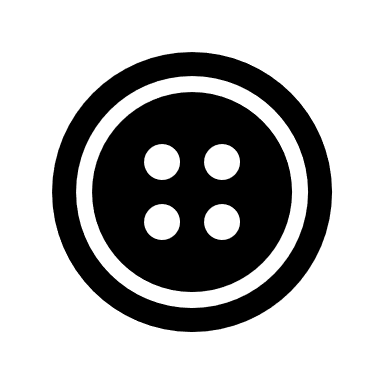

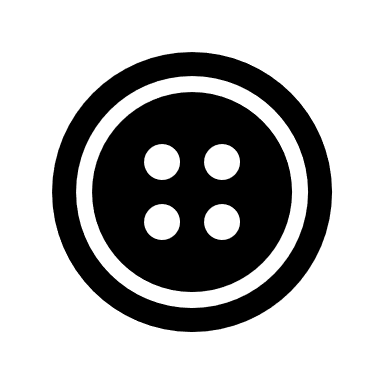

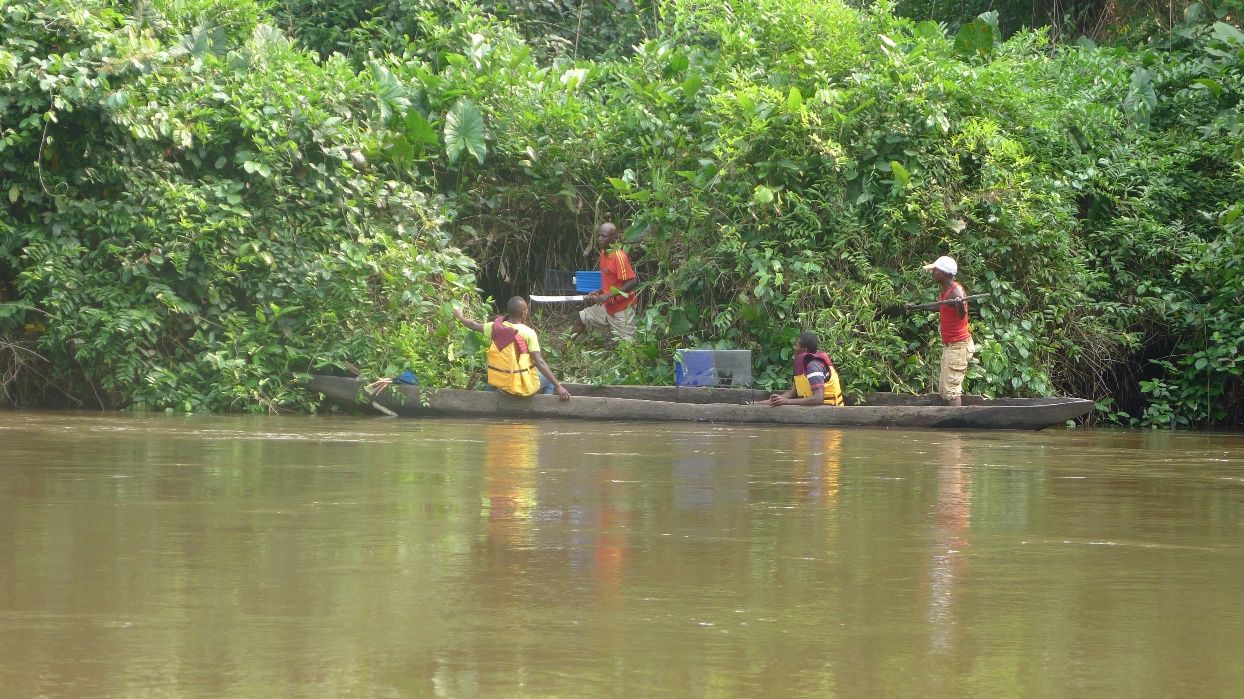


Fig C. Sensitization material in Kikongo

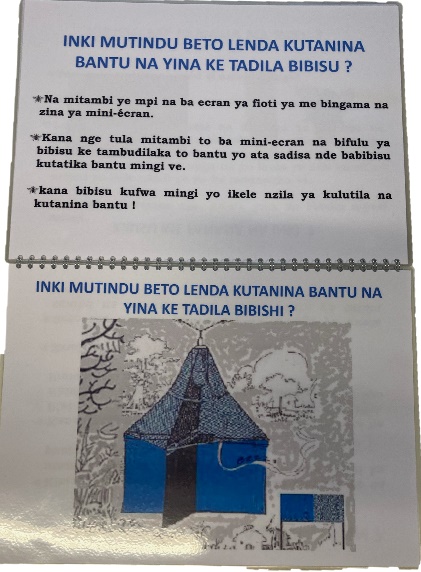

Table A. Calendar of vector control activities between 2015 and 2017

Table B. Details Financial costs

| **Description / cost category** | | **Total (USD) Y1** | **Total (USD) Y2** | **Total (USD) Y3** | **Total (USD) Y4** | **Total (USD) Y5** |
| --- | --- | --- | --- | --- | --- | --- |
| **Monitoring/Surveillance** | | **14,214** | **14,214** | **14,214** | **14,214** | **14,214** |
|  | HR | 2,610 | 2,610 | 2,610 | 2,610 | 2,610 |
|  | Transport | 5,443 | 5,443 | 5,443 | 5,443 | 5,443 |
|  | Traps | 4,189 | 4,189 | 4,189 | 4,189 | 4,189 |
|  | Other | 1,972 | 1,972 | 1,972 | 1,972 | 1,972 |
| **Sensitization** | | **44,217** | **19,541** | **27,717** | **19,541** | **27,717** |
|  | HR | 5,775 | - | 5,775 | - | 5,775 |
|  | Transport | - | - | - | - | - |
|  | Specialized equipment | 30,159 | 11,670 | 13,659 | 11,670 | 13,659 |
|  | Other | 8,283 | 7,871 | 8,283 | 7,871 | 8,283 |
| **Biannual target deployment** | | **57,571** | **57,571** | **57,571** | **57,571** | **57,571** |
|  | Tiny Targets | 26,399 | 26,399 | 26,399 | 26,399 | 26,399 |
|  | HR | 14,016 | 14,016 | 14,016 | 14,016 | 14,016 |
|  | Transport | 10,198 | 10,198 | 10,198 | 10,198 | 10,198 |
|  | Other | 6,959 | 6,959 | 6,959 | 6,959 | 6,959 |
| **Management support costs** | | **52,476** | **13,305** | **13,780** | **13,755** | **19,286** |
|  | HR | 10,345 | 10,345 | 10,345 | 10,345 | 10,345 |
|  | Transport | 35,227 | 2,487 | 2,487 | 2,487 | 2,487 |
|  | Specialized equipment | - | - | - | - | - |
|  | Other | 1,398 | 473 | 948 | 923 | 948 |
|  | Trainings/meetings | 5,506 | - | - | - | 5,506 |
| **Total cost** | | **168,478** | **104,630** | **113,282** | **105,080** | **118,788** |

Details Economic costs

**Table C. Deployment costs**

| Deployment costs | | |
| --- | --- | --- |
| 5 deployments (August '15 -Sep '17) | | |
|  | Number of Targets: | 39,198 |
| **cost category** | **Field Costs (USD)** | **%** |
| Tiny Targets with Vestergaard | 45,743 | 46 |
| Traps purchased locally | - | 0 |
| HR | 24,285 | 24 |
| Transport | 17,670 | 18 |
| Other | 12,058 | 12 |
| Total | 99,755 |  |
|  |  |  |
| Top-Down vector control - Deployment costs | | |
| Cost per target deployed => Total cost / number of targets deployed | | |
|  | # Targets: | 1 |
| **cost category** | **Field Costs (USD)** | **%** |
| Tiny Targets with Vestergaard | 1.17 | 46 |
| Traps purchased locally | - | 0 |
| HR | 0.62 | 24 |
| Transport | 0.45 | 18 |
| Other | 0.31 | 12 |
| Total | $2.5 |  |

|  |  |  |
| --- | --- | --- |
|  |  |  |
| Details per deployment | | |
| Jul-Sep'15 (August '15) | # Targets: | 4,782 |
| **cost category** | **Field Costs** | **%** |
| Tiny Targets with Vestergaard | 5,580 | 30 |
| HR | 3,867 | 21 |
| Transport | 5,801 | 32 |
| Other | 3,071 | 17 |
| Duration - Months: 1 | Cost per Target deployed | 3.8 |
|  |  |  |
| Jan-Feb '16 | # Targets: | 4,366 |
| cost category | Field Costs | % |
| Tiny Targets with Vestergaard | 5,095 | 32 |
| HR | 5,456 | 35 |
| Transport | 3,849 | 25 |
| Other | 1,287 | 8 |
| Duration - Months: 2 | Cost per Target deployed | 3.6 |
|  |  |  |
| Jul-Sep'16 | # Targets: | 10,947 |
| **cost category** | Field Costs | % |
| Tiny Targets with Vestergaard | 12,775 | 56 |
| HR | 4,866 | 21 |
| Transport | 1,253 | 5 |
| Other | 3,954 | 17 |
| Duration - Months: 2 | Cost per Target deployed | 2.1 |

|  |  |  |
| --- | --- | --- |
| Jan-Feb '17 | # Targets: | 7,792 |
| **cost category** | Field Costs | % |
| Tiny Targets with Vestergaard | 9,093 | 64 |
| HR | 833 | 6 |
| Transport | 2,895 | 20 |
| Other | 1,449 | 10 |
| Duration - Months: 2 | Cost per Target deployed | 1.8 |
|  |  |  |
| Jul-Sep'17 | # Targets: | 11,311 |
| **cost category** | Field Costs | % |
| Tiny Targets with Vestergaard | 13,200 | 46 |
| HR | 9,263 | 32 |
| Transport | 3,872 | 14 |
| Other | 2,296 | 8 |
| Duration - Months: 2 | Cost per Target deployed |  |

**Table D. Monitoring and surveillance**

| Monitoring Costs | | |
| --- | --- | --- |
| jan '15 - September 2017 - 33 Months | | |
|  | **Months:** | **33** |
| **cost category** | **Field Costs USD** | **%** |
| Reusable traps purchased from Vestergaard | 6,096 | 18 |
| Reusable traps purchased locally | 5,423 | 16 |
| HR | 7,178 | 21 |
| Transport | 14,969 | 44 |
| Other | 5,423 | 16 |
|  | 39,089 |  |
|  |  |  |
| Annual Monitoring Costs | | |
| Cost per year => Total cost / number of months => Multiplied x 12 | | |
|  |  |  |
| **cost category** | **Field Costs USD** | **%** |
| Reusable traps purchased from Vestergaard | 2,217 | 7 |
| Reusable traps purchased locally | 1,972 | 6 |
| HR | 2,610 | 8 |
| Transport | 5,443 | 16 |
| Other | 1,972 | 6 |
|  | 14,214 |  |

|  |  |  |
| --- | --- | --- |
|  |  |  |
| Details per monitoring & surveillance period | | |
| **Jan '15 -Sep - '15** |  |  |
| **cost category** | **Field Costs USD** | **%** |
| Specialized equipment | 500 | 14 |
| HR | 422 | 12 |
| Transport | 1,717 | 49 |
| Other | 891 | 25 |
| Duration - Months: 8 | 3,531 |  |
|  |  |  |
| **Oct '15 - Feb'16** |  |  |
| **cost category** | **Field Costs USD** | **%** |
| Specialized equipment | - | 0 |
| HR | 1,023 | 30 |
| Transport | 1,227 | 36 |
| Other | 1,206 | 35 |
| Duration - Months: 5 | 3,455 |  |
|  |  |  |
| **Mar-Sep'16** |  |  |
| **cost category** | **Field Costs USD** | **%** |
| Specialized equipment | - | 0 |
| HR | 3,553 | 37 |
| Transport | 5,534 | 57 |
| Other | 559 | 6 |
| Duration - Months: 7 | 9,645 |  |

|  |  |  |
| --- | --- | --- |
| **Oct -Feb '17** |  |  |
| **cost category** | **Field Costs USD** | **%** |
| Specialized equipment | - | 0 |
| HR | 634 | 16 |
| Transport | 2,942 | 76 |
| Other | 286 | 7 |
| Duration - Months: 5 | 3,862 |  |
|  |  |  |
| **March-Sep'17** |  |  |
| **cost category** | **Field Costs USD** | **%** |
| Specialized equipment | 4,924 | 47 |
| HR | 1,546 | 15 |
| Transport | 3,548 | 34 |
| Other | 549 | 5 |
| Duration - Months: 7 | 10,567 |  |

**Table E. Sensitisation cost**

| **Yasa Bonga:** |  | **165** | villages | |  |  |
| --- | --- | --- | --- | --- | --- | --- |
| **Description** | **cost category** | **Quantity** | **Unit cost (USD)** | **Total (USD)** | **Lifespan (in years)** | **Annual cost (USD)** |
| **Sensitization Training - Biennial** |  |  |  | **5,445** |  | **2,723** |
| Village leader | Other | 165 | 10 | 1,650 | 2 | 825 |
| Community health workers (CHW) | Other | 330 | 10 | 3,300 | 2 | 1,650 |
| Cost meeting – soft drinks- 1500 FC/1$ | Other | 495 | 1 | 495 | 2 | 248 |
| **Sensitization meeting - Evaluation - Biennial** |  |  |  | **5,445** |  | **2,723** |
| Village leader 1 per village | Other | 165 | 10 | 1,650 | 2 | 825 |
| CHW on average 3 per village | Other | 330 | 10 | 3,300 | 2 | 1,650 |
| Cost meeting – soft drinks- 1500 FC/1$ | Other | 495 | 1 | 495 | 2 | 248 |
| **Sensitization work/Follow up by community health workers Biennial** |  |  |  | **5,775** |  | **2,888** |
| CWH + village leader - 1 day | HR | 1,155 | 5 | 5,775 | 2 | 2,888 |
| **Material/equipment** |  |  |  | **32,997** |  | **18,596** |
| Stationery | Other | 1155 | 2.1 | 2,426 | 1 | 2,426 |
| Diffusion Spot radio - 6 times / day – 1 month | Specialized equipment | 1 | 120 | 120 | 1 | 120 |
| Th-shirts (CWH + village leader) | Specialized equipment | 1,155 | 10 | 11,550 | 1 | 11,550 |
| Development radio spot - per health district | Specialized equipment | 1 | 200 | 200 | 2 | 100 |
| Sensitization form (CWH + village leader) | Specialized equipment | 1155 | 0.12 | 139 | 2 | 69 |
| Megaphones | Specialized equipment | 165 | 10 | 1,650 | 2 | 825 |
| \|Batteries | Other | 165 | 2.5 | 413 | 2 | 206 |
| Sensitization picture box in Kikongo | Specialized equipment | 1155 | 10 | 11,550 | 5 | 2,310 |
| Banner Kikongo | Specialized equipment | 165 | 30 | 4,950 | 5 | 990 |
| **Total cost** |  |  |  | **49,662** |  | **26,929** |

**Table F. Management cost**

| **Unit Vector Control - Provincial level** | | **165** | villages | **4** | **Health districts** | |
| --- | --- | --- | --- | --- | --- | --- |
| **Category** | **Description** | **Unit price**  **(USD)** | **Life Expectancy  (years)** | **# Units annually** | **Annual cost**  **(USD)** | **Cost per HZ**  **(USD)** |
| HR | Anthropologist - Annual salary | 5,400 | NA | 1 | 5,400 | 1,350 |
| HR | Vector Control manager- Annual salary | 4,560 | NA | 1 | 4,560 | 1,140 |
| HR | Vector Control technicians - Annual salary | 3,600 | NA | 2 | 7,200 | 1,800 |
| HR | Driver- Annual salary | 3,600 | NA | 2 | 7,200 | 1,800 |
| HR | Supervision Coordination | 630 | NA | 4 | 2,520 | 630 |
| HR | Per diem anthropologist (10 days /month) | 25 | NA | 120 | 3,000 | 750 |
| HR | Per diem VC technicians | 25 | NA | 140 | 3,500 | 875 |
| HR | Per diem VC Manager | 25 | NA | 60 | 1,500 | 375 |
| HR | Per diem driver | 25 | NA | 260 | 6,500 | 1,625 |
| Transport | Fuel (500 l/trimester) - 1.80 US$/liter | 900 | NA | 8 | 7,200 | 1,800 |
| Other | Laptop | 900 | 3 | 2 | 600 | 150 |
| Other | Working costs (stationery, paper, etc.) by trimester | 450 | NA | 4 | 1,800 | 450 |
| Transport | Vehicle | 65,481 | 5 | 2 | 26,192 | 6,548 |
| Other | Raincoat | 15 | 1 | 6 | 90 | 23 |
| Other | Tent | 85 | 2 | 2 | 85 | 21 |
| Other | Camping Mattress | 17 | 2 | 6 | 51 | 13 |
| Trainings/meetings | Entomology Training 2020 in Kikwit | 3,171 | 4 | 1 | 793 | 198 |
| Trainings/meetings | GIS Training in Kikwit | 10,334 | 4 | 1 | 2,583 | 646 |
| Trainings/meetings | Project Management Training | 8,519 | 4 | 1 | 2,130 | 532 |
| Transport | Change of tyres once annually | 246 | NA | 8 | 1,968 | 492 |
| Transport | Change of battery annually | 140 | NA | 2 | 280 | 70 |
| Transport | Car insurance | 250 | NA | 2 | 500 | 125 |
|  |  |  |  | **Total** | **85,652** | **21,413** |

| **Unit Vector Control - Central level** | | **165** | villages | **11** | **Health districts** | |
| --- | --- | --- | --- | --- | --- | --- |
| **Category** | **Description** | **Unit price (USD)** | **Life Expectancy  (years)** | **# Units annually** | **Annual cost (USD)** | **Cost per HZ (USD)** |
| HR | VC Data Manager - Annual salary | 6,600 | NA | 1 | 6,600 | 600 |
| HR | Vector Control manager- Annual salary | 6,600 | NA | 2 | 13,200 | 1,200 |
| HR | Vector Control Logistician- Annual salary | 4,620 | NA | 1 | 4,620 | 420 |
| HR | Driver- Annual salary | 3,660 | NA | 1 | 3,660 | 333 |
| Other | Working costs (stationery, paper, etc.) by trimester | 250 | NA | 4 | 1,000 | 91 |
| Other | Supervision Direction: Data management + VC | 4,295 | NA | 4 | 17,180 | 1,562 |
| Transport | Vehicle | 65,481 | 5 | 1 | 13,096 | 1,191 |
| Other | Laptops | 700 | 3 | 4 | 933 | 85 |
| Trainings/meetings | Action Plan Workshops | 10,256 | NA | 1 | 10,256 | 932 |
| Trainings/meetings | Annual Review meeting | 12,160 | NA | 1 | 12,160 | 1,105 |
|  |  |  |  | **Total** | **82,705** | **4,306** |
