# Supplementary material for: The cost of sleeping sickness vector control in Yasa Bonga, a health district in the Democratic Republic of the Congo
Source: PLoS Negl Trop Dis. 2024 Nov 21;18(11):e0011959. doi: 10.1371/journal.pntd.0011959 (PMC11666030; doi:10.1371/journal.pntd.0011959)
Supplement: Attachment — Submitted filename: ResubmissionLetter.pdf [file pntd.0011959.s003.pdf]

INSTITUTE OF TROPICAL MEDICINE ANTWERP

Rian Snijders
Department of Public Health
Unit of Epidemiology and Control of Tropical Disease
Nationalestraat 155
2000 Antwerp, Belgium
rsnijders@itg.be

TO: EDITORIAL OFFICE
PLOS NEGLECTED TROPICAL DISEASES

ANTWERP

31/08/2024

Subject: Resubmission of revised paper "The cost of sleeping sickness vector control in the Democratic Republic of the Congo “ (PNTD-D-24-00153)

Dear Editors and reviewers,

Thank you for your letter dated the 7th of August enclosing the reviewer’s comments. We have carefully reviewed all the feedback and remarks and revised the manuscript accordingly. The changes to the initial manuscript are shown in the track changes section of the Word document. Our detailed responses to the remarks of the reviewers are given in the annex of this letter.

We hope the reviewed version is now suitable for publication and look forward to hearing from you in due course.

Sincerely,

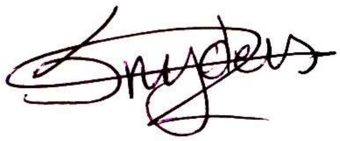

Rian Snijders

Annex: Response to reviewer

<u>Annex: Response to reviewer</u>
Please find our detailed responses to the remarks of the reviewers below in blue.

Methods
Reviewer #1: The objectives are very clear exposed and the study is adequate, but in my opinion there is an important mistake as it is not evident that the results obtained from only one district (Yasa Bonga) can be extrapolated to a whole country (DRC). This mistake is easily remediable changing the objective to estimate the costs of tsetse vector control to just to this district. In this sense, the title could be incorrect and should be modified to "the costs of sleeping sickness vector control in Yasa Bonga, DRC". This mistake should be also addressed in the abstract and discussion.
In this sense, the authors clearly state in the discussion (line 426-427). "In HAT endemic foci, adapting vector control strategies to local context is essential, introducing variability in the overall costs". DRC is a vast country with many variabilities between the different regions that make difficult the extrapolation from Yasa Bonga to the whole country.

Thank you for your constructive and positive feedback. We revised the title and discussion to highlight that this study focusses on the cost of vector control in one specific health district and not the entire DRC. The abstract and author summary already mentioned that the study assessed the cost of a health district.

Reviewer #2: The study presents a clearly articulated objective - costing tiny target deployment. Methods are described in detail. As the data being reported are actual rather than sampled programme costs, statistical considerations such as sample size and hypothesis tests are not required.

Thank you for the positive feedback.

Results

Reviewer #1: Taking into account the concern referred to the area of study (Yasa Bonga and not DRC), the results are very clear and the data provided in the supplementary files very completed.

Thank you for the positive feedback!

To include the details in the annexes makes easily readable and understandable the paper.
May be, it could be good to explain more clearly the difference between "targets used" and "targets deployed".

The difference between the 2 concepts is better explained in the “Cost methodology” section under material and methods.
Targets deployed refers to the number of targets set up in the area at a specific time, typically during a deployment cycle. This is a snapshot measure reflecting how many targets are physically in place to provide protection at any given time. While Annual Targets Used accounts for all the targets utilized throughout the year. Since targets are redeployed biannually, the annual target count will be higher, roughly double.

It is not clear how the area treated/covered by tsetse vector control has been calculated. I understand this aspect falls out of the purpose of this paper but it could be interesting to give some elements to know how it has been estimated. In fact. it looks like that there are areas considered as covered by vector control but in some of these areas, the closer Tiny Target is a more than 10 km (estimated from the map provided).

The calculation method of the area covered by vector control is explained in the material and methods section and the difference between Yasa Bonga and the other setting is explained in the results.

I don't know the region but authors refers that fishponds are widely distributed and can play a role in the presence of tsetse flies. Probably, there are also other small streams and tributary rivers on top of the three identified main rivers of the health district. Could they play a role in maintaining tsetse presence in theses areas?

A study by Tirados et al showed that secondary habitats in at-risk zones are generally connected to the river network and are considered part of interconnected tsetse fly populations. Analysis suggests that targeting the rivers, which are the highest-risk areas, should impact the associated secondary habitats as well, making rivers the priority for intervention. This information is also included in the discussion section.

Is the same method used in other regions used in the comparison of the cost?

The differences in tiny target deployment between the different regions is better explain in the revised text. Mandoul, with its vast and sparsely populated savannah, required widespread deployment over large areas with lower target density, making it more challenging and costly. Bonon located in a dense, forested region, allowed for concentrated target deployment in smaller, more accessible areas, resulting in greater efficiency. Arua, with its mix of savannah and forest, required a deployment strategy

targeting both terrain types. In Yasa Bonga, DRC, the remote, highly forested location posed significant logistical challenges, requiring intensive deployment strategies to ensure effective coverage. While Tiny Targets successfully reduced tsetse fly populations across all areas, the costs varied significantly depending on the criteria used for comparison.

For the cost methodology, the same methods were employed as with the other studies. Dr. Alex Shaw, who has been involved in all of the other costing exercises, is a co-author and played a supervisory role in part to be able to ensure comparability whenever possible. It is not always entirely possible to collect all the same data in all places, as sometimes the records differ a bit, but wherever possible, we made sure that we would account for all types of costs (fixed, capital, variable) costs in the same manner, and thus we provided Table 3 and the accompanying discussion to point out the reasons for any differences.

In graph 1 the different color between rivers covered starting 2015 and 2017 is not easily distinguishable. If possible, it is recommended to change the color.

The map was revised to better distinguish the colors between the scale up of vector control.

Reviewer #2: Results are clearly presented with appropriate detail.

Thank you for the feedback.

Conclusions

Reviewer #1: I have to acknowledge that when I received the paper, I was a bit misleaded by the title: I thought it will give an idea about how much is currently invested in tsetse vector control in DRC and how much will be needed to implement tsetse vector control, as recommended in the DRC, but this was not the case.

The title was revised clearly indicating that the article focuses on the cost of vector control at health district level in Yasa Bonga specifically.

It is not clearly stated in the paper but it looks like that vector control has been introduced in very limited health districts of the country (11 health districts and 2 provinces). How many health districts are considered endemic for HAT in DRC?. According to the paper used as reference 7, there are 507,000 square km at risk of HAT in DRC (2016-2020) with more than 42 million people living in this area. How many square km are currently covered by tsetse vector control?. Ideally, how many should be covered? According to the data obtained, which cost would have to cover all these areas? These figures will give really an idea of the cost of tsetse vector control in DRC as originally stated in the paper. This could be interesting to give an idea about the huge magnitude of the problem and the financial gap currently existing to reach the target of elimination of transmission, the limitations to extend the vector control activities, and why and how to prioritize vector control in some areas. This aspect is not considered in the discussion neither in the conclusions and it could be of high interest.

In March (after the initial submission of this paper) a preprint was published on the cost effectiveness of HAT eliminiation in the DRC estimating the cost to reach end of transmission. Results on the overall cost estimates for vector control are discussed in the discussion section of this paper.

Reviewer #2: A careful and insightful discussion is presented which highlights the interpretation and the limitations of the analysis. The discussion would benefit from a small amount of additional reflection on the importance of sensitisation activities to the effectivness of the intervention and the amount of time spent by community health workers supporting the intervention and whether this was or should have been costed.

In the Materials and Methods section, we provide a detailed explanation of the awareness-building strategy, which is currently factored into the cost analysis. Since this strategy was implemented after the study period, no data on its effectiveness is included. The cost estimates, along with a sensitivity analysis, account for the costs of sensitization, with a detailed breakdown available in the annex. Notably, sensitization activities constitute 22% of the overall annual cost, and the sensitivity analysis indicates that altering the frequency of these activities could significantly impact the annual budget.

Editorial and Data Presentation Modifications?

Reviewer #1: Title:

- Specify that the study is in Yasa Bonga and not all the country (DRC).

The title was revised “The cost of sleeping sickness vector control at health district level in Yasa Bonga in the Democratic Republic of the Congo”

Abstract:
- Specify that the study is in Yasa Bonga and not all the country (DRC).

The abstract was revised to include the name of the health district.

- You refer that the disease resulted in half a million deaths in the late twentieth century. This data is not shown in the introduction of the paper (you just refer thousands of people), so please include it also in the text with the adequate reference.

This information is also included in the introduction with reference.

Introduction:
- Lines 76-81: Please provide a reference for the first statement in the second paragraph ("By 1960,... and economic impact on the affected regions.").

A reference was added to the text.

- Lines 85-88: In the first statement of the third paragraph you refer to the "international community". This is quite vague. Please could you specify and provide a reference for this statement.

This statement was revised.

- Lines 135-139: May be to complete the paragraph, please indicate in how many districts and provinces tsetse vector control is currently used in DRC. Later on (lines 212-213) you refer that vector control management covers "11 health districts in 2 provinces". How many provinces and health districts are at this time considered as endemic for HAT?

This question is addressed in the discussion section where we included information on the coverage and cost of vector control needed to reach end of transmission by 2023.

- Line 139: Specify the area of study.

Name of the health district was added.

Material and methods:
- Line 172-173: I don't understand the conclusion stated as "... so that almost the whole district was protected". Checking the map provided, if I'm not wrong, it looks like 15 health areas were considered as "covered by vector control" but it looks like the district has 22 health areas. So 15/22 areas of the health district were covered by vector control. How do you consider 15/22 as almost the whole health district ?

This is revised in the article to around 70% of the district was protected.

- Line 209-210: You refer that 165 villages (over 305 villages of the district) were identified for sensitization. May be it could be interesting to give a short explanation about how these villages were identified.

This was revised Materials and Methods section.
In the health district, 165 villages close to the river borders that would be exposed to gHAT vector control as their population would see the Tiny Targets or deployment activities were identified for sensitization

Results:
- Table 3: Note that the comparison is between 4 specific and well defined endemic areas from 4 different countries. It will be more precise to indicate in the title row "Mandoul, Chad" (instead of "Chad"), "Bonon, Côte d'Ivoire" (instead of Côte d'Ivoire"), "Arua, Uganda" (instead of "Arua) and "Yasa Bonga, DRC" (instead of "DRC").

This is revised in the article

Discussion:
- Line 479: May be better "2017-2018" instead of "'17-'18"

This is revised in the article

References:
- There is a typo with reference 14, as lines are cut in different paragraphs.

This is revised in the references.

Reviewer #2: see above re sensitisation and community health worker role.

This is revised in the Materials and Methods section.

Summary and General Comments

Reviewer #1: Interesting paper, easily readable with clear results and discussions. The details in calculations are particularly good to understand the methods used and to do similar exercises. In my opinion, it includes an important mistake as it considers that the data obtained are applicable to DRC, when there were obtained just in one specific health district (Yasa Bonga) and it is no evident that this can be extrapolate to the whole country. I would strongly suggest that the paper is focusing just in this district and later in the discussion consider the value of the data for the whole country. In this sense, a most appropriate title could be "the cost of sleeping sickness vector control in the health district of Yasa Bonga, DRC" and to introduce the adequate changes in the abstract and in the discussion. This should be on line with previous similar articles, referred specifically to Arua and not Uganda (ref 17 and 20), to Bonon and not Côte d'Ivoire (ref 31 and 19), Mandoul and not Chad (ref 32 and 21). I think it is very important to include this consideration in the Title and Abstract of the paper.

This comment is addressed throughout the paper indicating the cost estimates focus on 1 health district in the DRC. In the discussion we included data from a recently published paper on the coverage and cost of vector control in the DRC to achieve end of transmission by 2030.

Reviewer #2: This study provides useful information for health economists and policy makers looking to understand the likely costs of vector control and what drives these costs. The information is provided in sufficient detail for those in other settings to adjust costs to their contexts. With minor adjustments to the discussion to touch on the importance of sensitisation to the effectiveness of vector control, and the potential burden on community health workers and how this was costed, I am happy to recommend this for publication.

Thank you for the recommendation
